# Bedside teaching – from clinical learning to explicit teaching

**DOI:** 10.3205/zma001850

**Published:** 2026-04-15

**Authors:** Thomas Rotthoff

**Affiliations:** 1 University of Augsburg, Faculty of Medicine, Medical Didactics and Education Research, DEMEDA, Augsburg, Germany

## Editorial

“Good morning, everyone. Welcome to bedside teaching. Please go to room 12 and take a medical history. We will then discuss the patient.” This is how lessons at the bedside often begin at German university hospitals: a brief introduction and a task, followed by the students disappearing into the patient’s room and the doctors into the doctors’ room or to attend to other patients. The students gain experience but mostly without direct supervision, structured observation, or situational feedback. Viewed objectively, what remains is the quiet hope that professional perception, clinical judgment, and the ability to take a relevant medical history and to recognize pathological findings will develop incidentally, so to speak; that clinicial judgement will be derived from the findings collected and that medical competence will ultimately be formed from this.

Things used to be different. In the 17^th^ century, teaching at the bedside was established as an integral part of medical studies at Leiden University. While Wilhelm Boerhaave was known for his focus on understanding and tracking clinical signs, Franciscus Sylvius paved the way for clinical teaching that was interactive and practical, with a focus on observing symptoms together, taking medical histories, and conducting physical exams [1], [2], [3]. William Osler then took up this principle programmatically in the 19th century: “Medicine is learned by the bedside and not in the classroom” [4]. Is this historical focus merely a nostalgic idealization of bedside teaching? Or does this tradition point to a lasting – perhaps even urgent – need to systematically and, above all, deliberately supervise the teaching and training of medical perception, medical history taking, and physical examination of patients?

Garibaldi and Russell recently explained in the New England Journal of Medicine that the declining amount of time available for teaching and learning at the bedside is having noticeable consequences: Key clinical skills – especially medical history taking and physical examinations – are increasingly falling by the wayside, and a significant proportion of diagnostic errors, especially in outpatient care, can be attributed to deficits in these basic skills [5]. This not only affects treatment outcomes but also causes additional costs. At the same time, confidence in technical examinations is growing, and where clinical certainty is lacking, diagnostic equipment is used more frequently; however, this is not always necessary and is often an expensive alternative. This creates a vicious cycle of dwindling competence and increasing overdiagnosis [5]. 

The necessity of clinical practical training in medical studies has been discussed in Germany for decades [6], [7] and is (still) embedded in the current German licensing regulations: 476 hours of bedside teaching, three months of nursing service, four months of clinical internship, clinical block internships, and finally, the so called “Practical Year” structure together the practice-oriented phase of medical studies [https://www.gesetze-im-internet.de/_appro_2002/BJNR240500002.html]. These formal requirements suggest substantial integration into clinical practice. But how much of this designated practice, actually consists of guided, didactically structured teaching – and how much of it is structured attendance as part of participation in everyday clinical work? 

A distinction must be made here: Not all attendance by students in a clinical context must didactically be framed as teaching, as work in clinics and outpatient clinics is primarily intended to provide patient care and not to serve as student learning. Students participate, observe, support, and take on tasks. A significant part of learning already takes place through such involvement in real care situations, since as educational scientist Wolfgang Sünkel put it, “there is no situation in human existence in which learning does not also take place” ([8], p.39). Learning is thus intertwined with the actual work, as students observe and acquire routines and attitudes through participation.

Bedside teaching with real patients, on the other hand, is a format that is specifically designed and didactically structured. This teaching forms a core part of clinical medical training and, according to the German Science Council, is characterized by continuous and direct observation by the teaching physician and differentiated feedback; concrete, actionable suggestions for improvement is provided to systematically develop the clinical skills of the students [7]. The German licensing regulations also explicitly refer to teaching and instruction when it comes to teaching with patients, whereas internships are merely described as learning opportunities in everyday clinical and outpatient medical practice [https://www.gesetze-im-internet.de/_appro_2002/BJNR240500002.html] and thus offer informal learning.

The primary goal of bedside teaching is therefore the targeted and guided development of professional competence. Such real teaching situations should always be created when activities or skills are so differentiated, complex, and difficult that they can no longer be learned with sufficient confidence and success through mere situational participation; learning must take place before competent action can be taken ([8], p.39). This form of learning is formal learning that is specifically organized and didactically guided. The clinical care activities of the physician must be “put on hold” during this time, and a change of perspective must take place from care (clinical thinking and action on the patient) to teaching (clinical thinking and action on the patient). A teaching situation only arises when the students, subject matter (and patient), and teacher interact; if one of these elements is missing, it cannot be considered teaching ([8], p.63). Formal and informal learning merge dynamically because, during patient-based teaching, students also observe the behaviour and skills of the teacher.

Bedside teaching as formal teaching thus creates a conflict situation because, in the complex world of medical care, doctors’ working hours do not allow them to switch between care and formal teaching at will ([8], p.48). The main and well-known obstacles include time pressure, staff shortages, and short patient stays. However, another aspect is often underestimated: Many teachers are not aware that bedside teaching is a didactically guided format that requires active structuring. Instead, it is often understood that students should try things out for themselves – which in practice then leads to a “go-ahead-and-do-it” approach. This attitude promotes a low level of didactic guidance and reinforces uncertainties on both sides. To make matters worse, bedside teaching is surrounded by a special aura: It is considered a teaching format in which teachers at the bedside must have exceptionally high diagnostic skills [9]. This expectation creates pressure on teachers to perform – and often has a deterrent rather than an encouraging effect. As a result, the format may be avoided or reduced to a minimum instead of being used as a structured learning space for joint clinical thinking and professional action. The described conflict situations point to a complex problem that cannot be solved in isolation. Bedside teaching fails not so much because of a lack of goodwill but rather because of a lack of structural support, insufficient didactic support, and a teaching culture that has not yet been established consistently. 

Bedside teaching in everyday clinical practice remains a challenge – but three key approaches could be adopted to help address this. First, bedside teaching requires clear structures with scheduled teaching times and clearly defined responsibilities. Selected areas of practice in which successful examples of implementation are developed would also be beneficial, as these can then be disseminated via stories of success and physicians who act as multipliers. Second, targeted didactic professionalization of the bedside teaching format through the application of basic training, structured instructions, and feedback and mentoring would improve the quality of teaching at the patient’s bedside. Third, the attitudes of leaders are crucial. If leaders are visibly present during bedside teaching and teaching performance with patients is valued and made relevant to career advancement, a common understanding will emerge that this task is a professional mission rather than an additional individual burden.

Several articles in this issue highlight the importance of simulations in medical education to prepare students for direct contact with patients. For example, Brotons de los Reyes et al. demonstrate the importance of simulations in preparing students for direct contact with patients by validating a tool for assessing empathy using simulated patients [10]. Saitta et al. compare high-fidelity and seminar-based simulation environments [11], Steinacker et al. describe perspective-changing exercises in nursing education [12], and Strumpski et al. describe a longitudinal communication curriculum in the context of dentistry [13]. These articles highlight various ways in which simulation can contribute to the development of professional skills. Both simulations and bedside teaching contribute to the development of clinical skills, but direct contact with patients remains the place where competencies are integrated and learning unfolds its full clinical significance.

## Notes

### Use of AI

The title of this editorial was generated by prompting in ChatGPT (OpenAI, version 5.2).

### Author’s ORCID

Thomas Rotthoff: [0000-0002-5171-5941]

## Competing interests

The author declares that he has no competing interests.
